# The effects of short term detraining and retraining on physical fitness in elite soccer players

**DOI:** 10.1371/journal.pone.0196212

**Published:** 2018-05-10

**Authors:** Chang Hwa Joo

**Affiliations:** Department of Football Science, Honam University, Gwangsan-gu, Gwangju, South Korea; Sao Paulo State University - UNESP, BRAZIL

## Abstract

**Purpose:**

The aim of this study was to examine the effects of aerobic high-intensity training with reduced volume and training cessation on body composition and physical fitness after the end of season and the time required to recapture physical fitness with intensified retraining following two weeks of detraining in elite soccer players.

**Method:**

Twenty male semi-professional soccer players participated in this study. The soccer players were assigned to either a group that completed high-intensity aerobic training (HAT, n = 10) or to a detraining and retraining group (DHAT, n = 10) for a 5-week period immediately after the end of the season. The first 2 weeks of the period, members of the HAT group performed high-intensity aerobic exercise (80–90% of HRmax, 12 min × 3, three times per week), whereas members of the DHAT group abstained from any physical activity. During the subsequent 3 weeks, members of both the HAT and DHAT groups completed high-intensity aerobic exercise. Exercise performance testing and body composition analysis were performed before; after 2 weeks of detraining; and at 1, 2 and 3 weeks of retraining.

**Results:**

Intensified high-intensity training for 5 weeks maintained the performance in the Yo-Yo Intermittent Recovery level 2 test (Yo-Yo IR2) and repeated sprints at any time point (*P* > 0.05). However 2 weeks of detraining resulted in significant decreases in the performance on the Yo-Yo IR2 (*P* < 0.01) and repeated sprints test (*P* < 0.05). Performance on the Yo-Yo IR2 enhanced after 2 weeks of retraining and was maintained up to 3 weeks after retraining, with no significant differences between conditions (*P* > 0.05). In addition, repeated sprint performance markedly decreased after the detraining period (*P* < 0.05) and was continuously lower compared to the baseline at 2 weeks after retraining (*P* < 0.05). Furthermore, this value reached baseline level at the end of the experimental period (*P* > 0.05). There were no significant differences between conditions in body composition, performance of agility, or sprint ability throughout the 5-week experimental period (*P* > 0.05).

**Conclusions:**

The present data suggest that short-term detraining after the competitive season can markedly decrease performances in the Yo-Yo IR2 test and repeated sprints. To return to a previous level of ability on the Yo-Yo IR2 and/or sprint test with retraining through high-intensity aerobic training after a period of detraining, a similar or longer period of retraining is required. However, the high-intensity training with reduced amount of training after competitive season can prevent reductions in physical fitness.

## Introduction

Soccer is a high intensity intermittent exercise that requires a high level of physical fitness for players to successfully perform in the game. Elite soccer players perform 587±133 m of high-speed running (19.8–25.2 km/h) and 184 ± 87 m of sprinting (>25.2 km/h) during a typical game [1]. The total distance of high-intensity running depends on the position of the player and team success in a league [2]. The amount of high-intensity running performed during a game also depends on the competitive standards between leagues: top-class professional soccer player perform more high-intensity running compared with moderate professional soccer players [3]. Thus, high level of physical performance is an important factor in determining team success in soccer.

Due to the high intensity performance required in soccer, players should perform systematic and scientific physical fitness training. Several studies have shown that high-intensity training improves soccer players’ fitness levels and skills, such as sprint, strength, and speed endurance [4, 5]. The organization of fitness training for soccer players varies according to the time frame of the periodization along with changes in training volume and intensity. These changes seek to the optimize player’s physical condition and minimize injury [6]. For example, training is conducted to improve physical fitness during the preseason in preparation for the impending competitive season [7, 8].

Elite soccer players normally cease training or perform training with reduced volume and lower intensity for more than two weeks after the end of the competitive season for physical and mental recovery. A prolonged period of rest after the competitive season causes the partial or complete loss of training-induced physiological and performance adaptations, which is defined as detraining [9]. The magnitude of changes during training-induced adaptations after detraining is different depending on the fitness level and the duration of training cessation or insufficient training [9]. Three to six weeks of detraining did not result in changes in aerobic capacity and muscle strength in recreational players and untrained individuals [10–12]. However, decreases in physical fitness are inevitable after such a period of detraining in well-trained elite players who have a relatively higher level of fitness compared to recreational players [9, 13]. Unlike reduced physical fitness after a prolonged period of detraining in elite players, the effects of short-term detraining (~2 weeks) on fitness are controversial. Buchheit et al. [14] observed that short-term detraining after a competitive season improved levels of strength and cardiorespiratory fitness in Australian football players [14]. In contrast, several studies reported that physical fitness was reduced after a short-term detraining period in elite soccer players [5, 15]. The reasons for these contrasting results are not apparent, but may be due to differences in sports and testing methods.

During the preseason, the aim of training is mainly to improve physical fitness, while during the in-season period, it is performed to develop playing strategies and to enhance performance, while maintaining physical fitness. High-intensity training is a more efficient method of inducing skeletal muscle adaptation in comparison to moderate-intensity training [16]. High-intensity aerobic training has been widely used by athletes to improve physical fitness during the preseason. Indeed, high-intensity aerobic training consisting of 4 bouts of 4 min at 90–95% of the maximum heart rate during the preseason significantly improved aerobic fitness and match performance in soccer players [17]. Those results indicated that high-intensity aerobic training might be effective at improving the physical fitness of soccer players and inducing rapid training adaptation in skeletal muscle during the preseason.

In order to start the season without injury, athletes must gradually improve their post-season, resting period-induced reduction in physical fitness with an appropriate exercise intensity and volume. However, there is limited information available regarding the effects of retraining during pre-season training in well-trained elite soccer players. Therefore, the aim of the study was to investigate 1) the effects of aerobic high-intensity training with reduced volume and training cessation on body composition and physical fitness after the end of season and 2) the time required to return to the previous level of physical fitness with intensified retraining following two weeks of detraining in semi-professional soccer players.

## Materials and methods

### Participants

Twenty semi-professional male Korea soccer players (age: 22.1±1.8 years, height: 175.5±4.7 cm). The Korean professional soccer league is divided into K League Classic (first division) and K League Challenge (second division). The semi-professional league consists of the National League and K3 Leagues (K3 League Advanced [12 teams] and K3 League Basic [8 teams]). The soccer players participating in this study were members of K3-league teams. All participants had experience of elite soccer players for at least more than 7 years. All participants were non-smokers, no history of neurological disease or musculoskeletal abnormality and none were under any pharmacological treatment during the course of the study.

### Ethics statement

Before testing, all participants gave written informed consent to participate after details and procedures of the study had been fully explained. All of the fitness testing and exercise were performed in the research institute for sport and exercise science at Honam Unviersity. All of the experimental protocols and related procedures were approved by the ethical committee of Honam University.

### Intervention period and training

All players participating in the study trained for more than 2 hours per day for 4–5 times per week (excluding matches) during the previous season. An independent research assistant selected the 20 participants from among 35 players who were between 20 and 23 years of age by drawing a sealed envelope containing a player’s name followed by drawing another sealed envelope containing the name of the group to which they were assigned (i.e., high-intensity aerobic training (HAT) or detraining and high-intensity aerobic training (DHAT) group). The two-week detraining period started immediately after the last match of the season. The fitness tests were conducted two days and one day before the last match as a pre-test; after two weeks of detraining; and at one, two, and three weeks of retraining.

**Fig 1:**
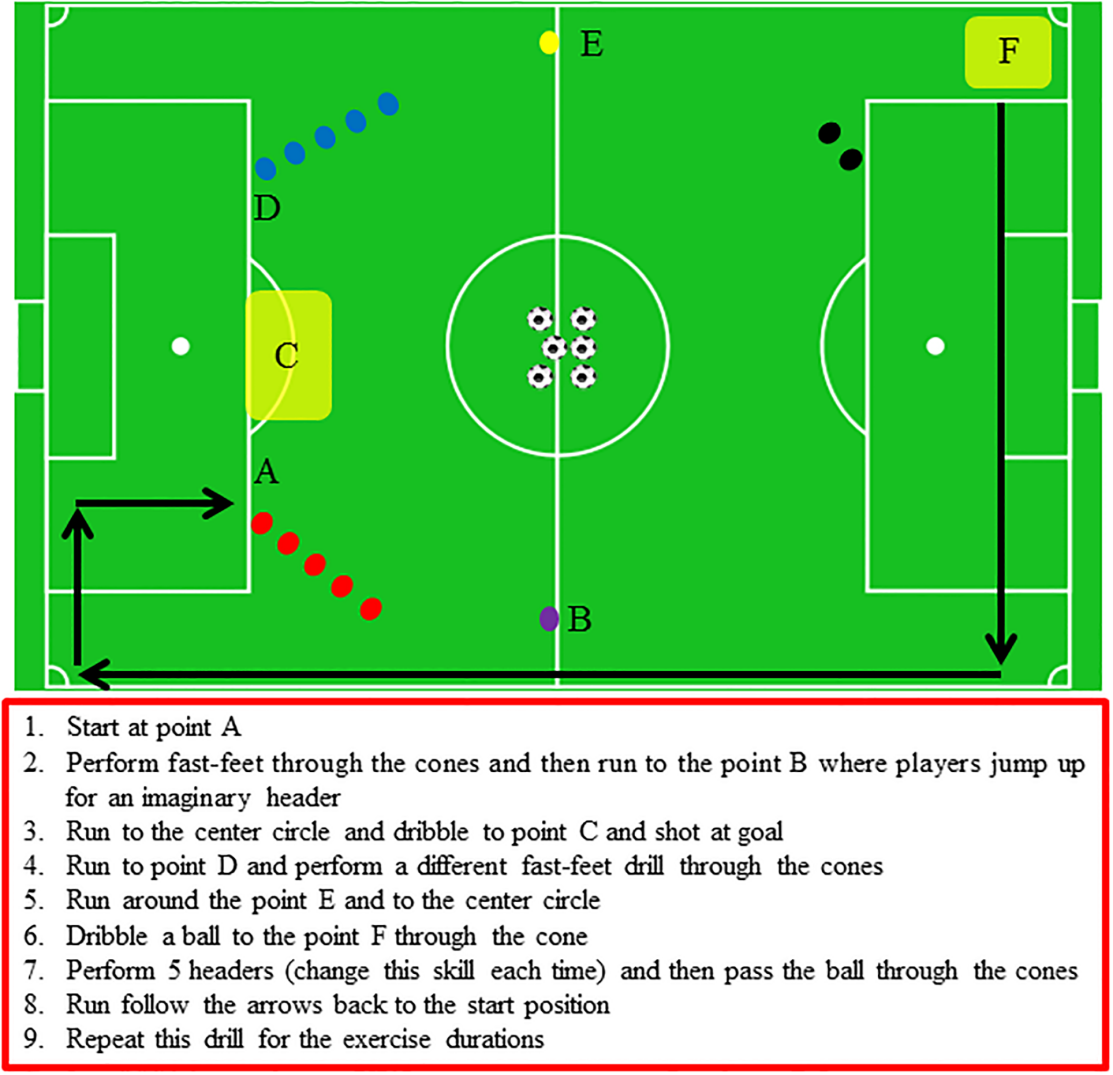
Diagram of high-intensity training.

During the detraining experimental period, high-intensity aerobic training was performed three times per week for two weeks in the HAT group. After approximately a 20-min warm-up period, the players performed a soccer drill (Fig 1) on an artificial grass surface and three repetitions of 12 min of exercise at 80–90% of the maximum heart rate (HRmax) measured during Yo-Yo IR2 test. These repetitions were interspersed by 3 min active recovery. The players controlled exercise intensity by watching their HR monitor that recorded at 5 s intervals (Polar Team System, Polar, Electro Oy, Kempele, Finland). These data were downloaded to a personal laptop for further analysis. The mean HR during the 12 min exercise sessions was 87.3±1.5% of HRmax. The DHAT group did not perform any exercise sessions during the two weeks of detraining and conducted normal daily activities.

During the retraining period, the HAT and DHAT groups performed high-intensity aerobic training (12 min × 3) four times per week for three weeks. The mean HR during the 12 min exercise sessions was 86.5±1.4% of HRmax in the HAT group. The DHAT group completed moderate intensity aerobic training (HRmax 70–80%; 76.5±3.2%) for two days before completing the high-intensity training (HRmax 80–90%; 87.7±1.3%).

### Experimental protocol

**Fig 2:**
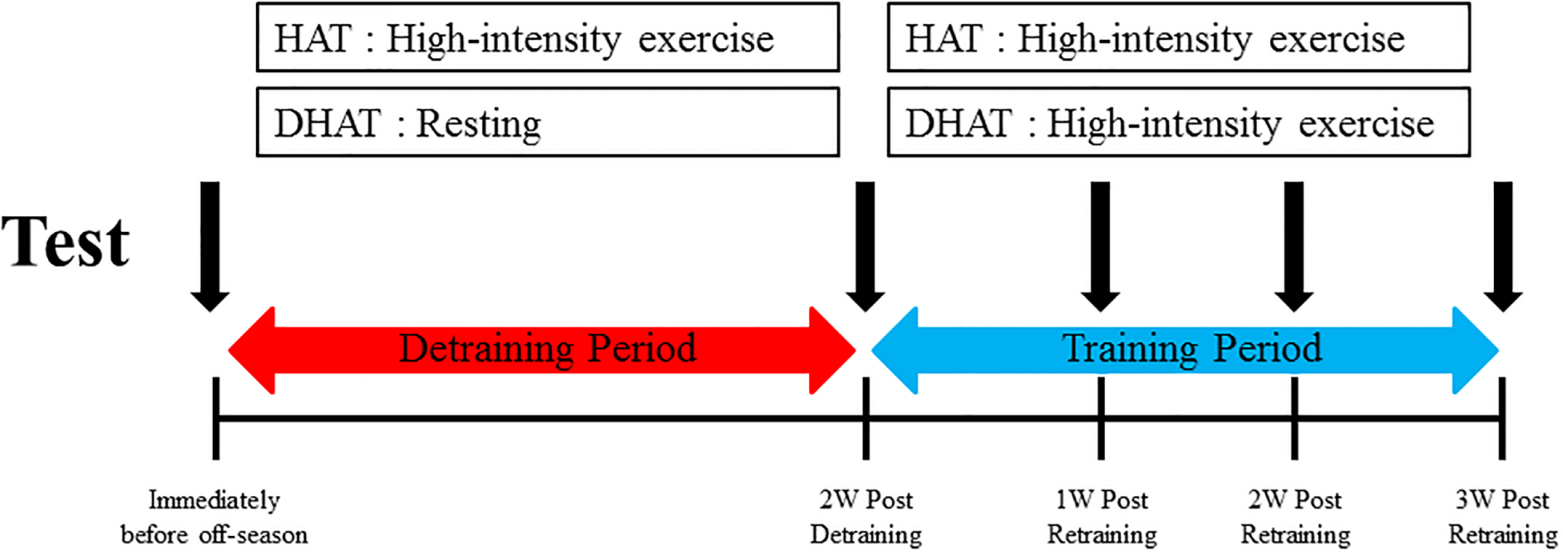
A schematic illustration of the experimental design.

A schematic illustration of the experimental design is shown in Fig 2. The subjects completed the 30 m sprint test, Yo-Yo intermittent recovery level 2 (Yo-Yo IR2) test, arrowhead agility test, repeated sprint test, and isokinetic strength test. The tests were conducted for two days. The participants refrained from alcohol and caffeine in the 24 h prior to the test. The participants arrived at the laboratory having completed the appropriate diet regime to monitor the diet level. The participants were instructed to ingest water 5 mL of water for every kilogram of their body mass 2 h before arriving at the laboratory. Upon the arrival at the laboratory, body composition (Inbody 520, Biospace, Seoul, Korea) and height (BSM, Seoul, Korea) were measured. Following the completion of the baseline assessments, the participants commenced the tests on an artificial grass surface. A 30-m sprint test, arrowhead agility test, and repeated sprints test were performed in the morning. The Yo-Yo IR2 test was conducted in the evening with 5 hours of recovery after lunch. Isokinetic strength tests were performed in the laboratory the next day. Body composition and exercise tests were completed immediately before the end of the season; after two weeks of detraining; and at one, two, and three weeks of retraining intervention.

### 30m sprint test

The sprint tests which consisted of 2 maximal sprints of 30 m with 2-minute rest between each sprint were conducted. The sprint times at 5, 10, 20 and 30 m were recorded using the photocell gates (Microgate, Bolzano, Itaia). The participants started to run 50 cm before the photocell gate recordings. The fastest times at the distances were recorded for data analysis.

### Repeated sprint test

The repeated sprint test consisted of seven maximal 34.2 m sprints, interspersed by 25 s of active recovery (40 m jogging distance) [18]. Recovery was timed so that the subjects returned to the start line between the 23^rd^ and 24^th^ second. Additionally, verbal feedback was given at 5, 10, 15 and 20 s of the recovery. Performance was measured as the total sprint time in seconds.

### Yo-Yo intermittent recovery test (level 2)

The Yo-Yo IR2 test was performed on an artificial turf. The Yo-Yo IR2 test consists of 2 × 20 m shuttle runs at increasing speeds, controlled by audio signals from a compact disk. Between each bout of running, the subjects completed 10 s of active recovery, consisting of 2 × 5 m jogging [19]. The test was terminated when the subjects failed twice to reach the start line on time and the distance (meters) covered at the end point was recorded [5].

### Arrowhead agility test

The arrowhead agility tests consisted of 4 sprints (2 right, 2 left), with 2-minutes rest between each sprint [20]. Each subject started 50 cm behind the start line and sprinted 10 m forward to a cone. From the cone, the subjects turned at a right angle to a cone being apart from 5m before turning to a cone 15 m straight from the start line. They turned again from the cone to accelerate in a straight line for 15 m over the initial start line to complete the run. The fastest times were recorded for data analysis. Timing gates were used to accurately assess the time to completion.

### Isokinetic strength

The subjects performed the Isokinetic dynamometry (Cybex MET-300, New York, USA) to evaluate the unilateral strength of the concentric contraction of the flexors and extensors of the knee [21]. The angular speed parameters of 60° × s^-1^, 180° × s^-1^, and 240° × s^-1^ were used for the measurements. The results of the measurements were expressed in absolute peak torque (Nm) for the purposes of the off-seasonal variation comparisons.

### Statistical analysis

All data are presented as means ± SD. Two-way analysis of variance (ANOVA) with repeated measure was conducted to determine any treatment differences between the HAT and DHAT conditions. The assumption of sphericity (homogeneity of covariance) was assessed and corrected for using the Huynh-Feldt epsilon. Because there were only 2 levels in the main effect of condition, follow-up multiple comparisons were not necessary. A significant effect of time was followed up with planned multiple contrasts in line with the a priori hypotheses. Therefore, data at the specific time points were compared with the baseline (first) time point using Newman-Keuls multiple contrasts. Where a significant interaction between condition and time was observed, differences between conditions were examined at each time point using Newman-Keuls multiple contrasts. Baseline values were compared using an independent samples *t* test. The alpha level for evaluation of statistical significance was set at *P* < 0.05. Effect sizes were assessed by partial eta squared ($\eta_{P}^{2}$), which were defined as trivial (<0.1), small (0.1–0.3), moderate (0.3–0.5) and large (>0.5) [22].

## Results

**Fig 3:**
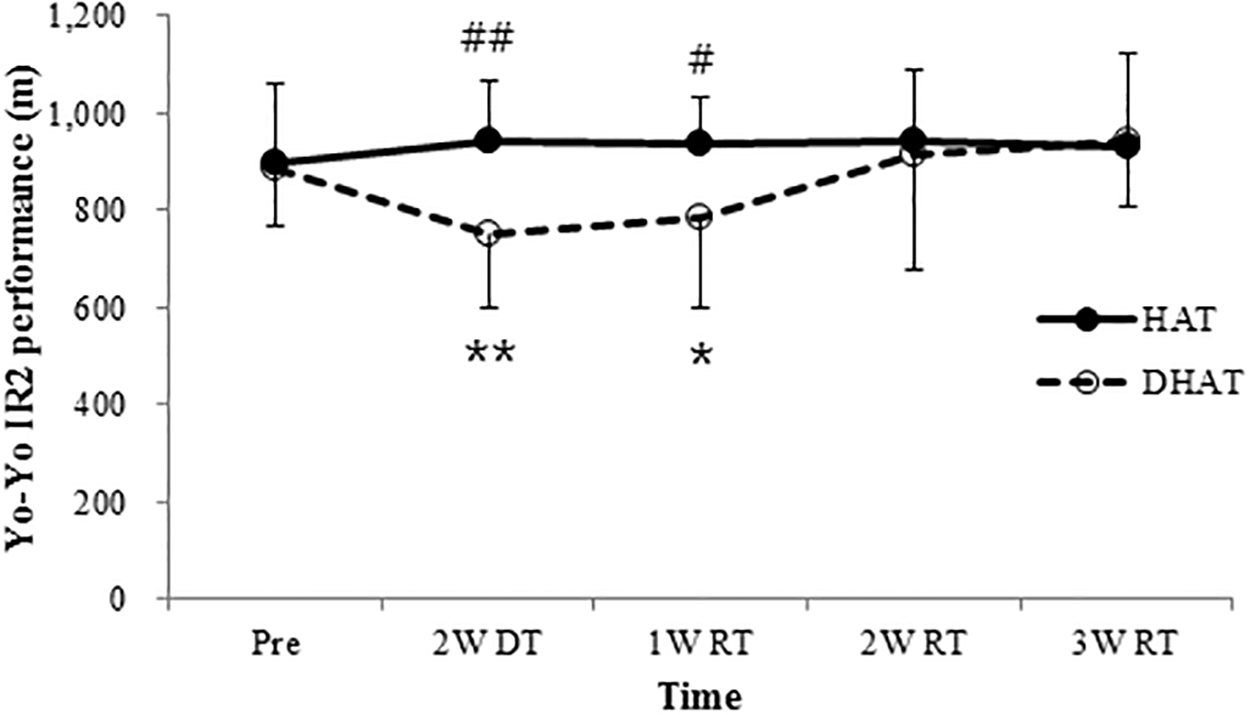
Yo-Yo IR2 performan for the high-intensity training (HAT; n = 10) and detraining + retraining (DHAT; n = 10) before, after two weeks detraining and at one, two and three weeks of retraining (n = 11, mean ± SD). **P < 0.01; significantly different from pre. *P < 0.05; significantly different from pre. ##*P* < 0.01; significantly between conditions. #*P* < 0.05; significantly between conditions.

**Fig 4:**
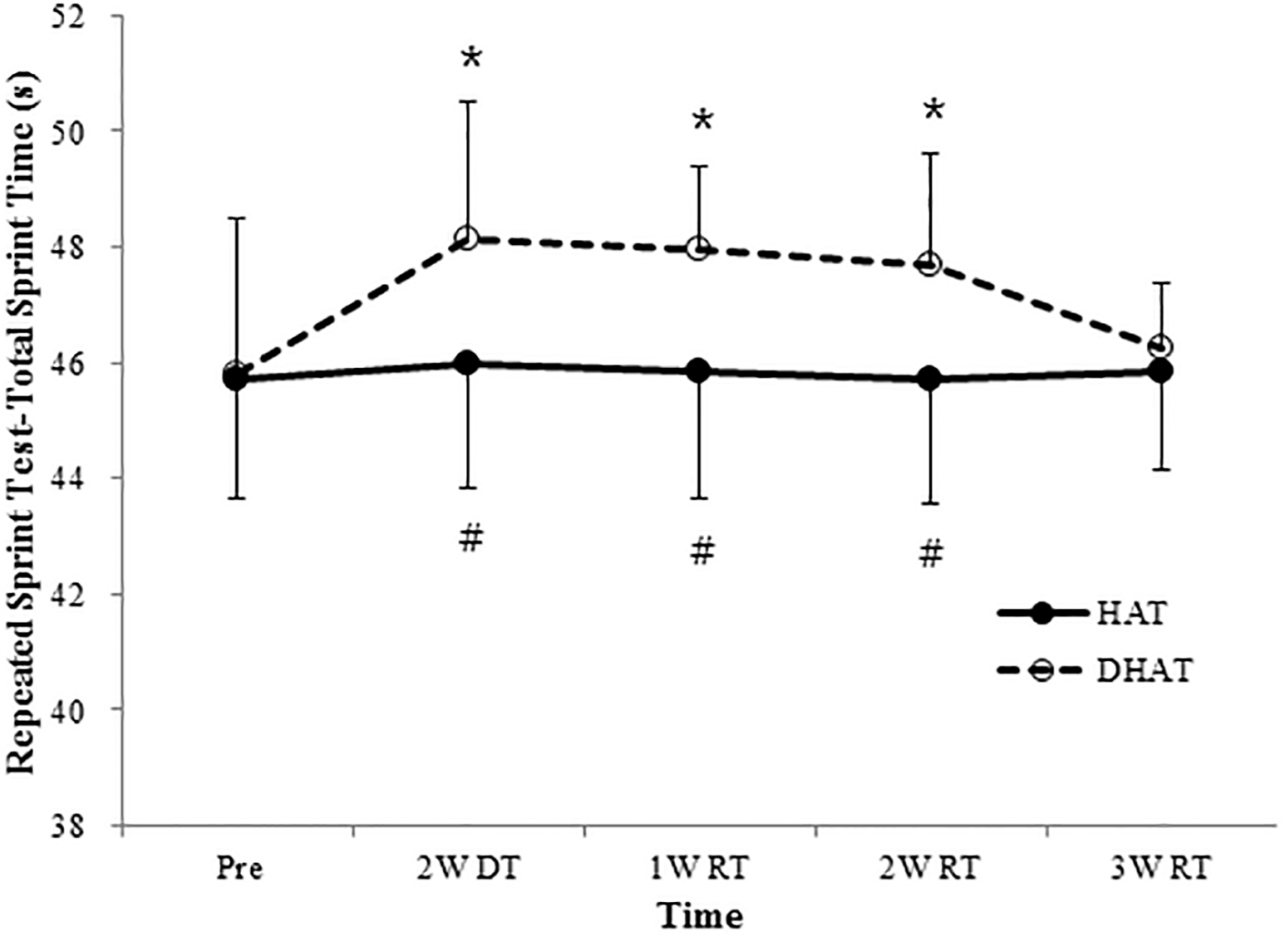
Repeated sprint test for the high-intensity training (HAT; n = 10) and detraining + retraining (DHAT; n = 10) before, after two weeks detraining and at one, two and three weeks of retraining (n = 11, mean ± SD). *P < 0.05; significantly different from pre. #*P* < 0.05; significantly between conditions.

Body weight and body fat were similar between the HAT and DHAT groups throughout the experimental period (*P* > 0.05; Tables 1 and 2). There was no significant effect of condition nor was there an interaction of condition and time (*P* > 0.05) in the performance of players on sprint and agility tests (Tables 3 and 4). However, a significant effect of time was observed for sprint test at 5 m, 10 m, and 30 m as well as in the left direction of arrowhead agility (*P* < 0.05). Isokinetic strength at all angular speeds remained similar to baseline under both conditions throughout the experimental period, with no significant effects of time, condition, or an interaction between the two (*P* > 0.05; Tables 5 and 6). There was a significant interaction in the Yo-Yo IR2 test (*F* = 3.273; *P* < 0.05; $\eta_{P}^{2}=0.267$), while the measurement time (*F* = 1.517; *P* > 0.05; $\eta_{P}^{2}=0.144$) and condition were not significant (*F* = 1.938; *P* > 0.05; $\eta_{P}^{2}=0.177$). Compared to the pre-detraining performance, the Yo-Yo IR2 test performance decreased significantly after the two-week detraining period (*P* < 0.01) and the values reach before detraining level after two weeks of retraining in the DHAT group (*P* > 0.05). No differences were detected at three weeks post-retraining between conditions (P > 0.05), whilst values in the HAT group remained stable throughout the experimental period (*P* > 0.05; Fig 3). A main effect of time was found (*F* = 3.539; *P* < 0.05; $\eta_{P}^{2}=0.282$), along with a significant interaction between condition and time for repeated sprint performance (*F* = 3.127; *P* < 0.05; $\eta_{P}^{2}=0.258$). No changes in repeated sprint performance were observed at any time point under HAT conditions (*P* > 0.05), whereas repeated sprint performance declined after two weeks of detraining (*P* < 0.05) and remained lower than at baseline by two weeks post-retraining under DHAT conditions (*P* < 0.05). It reached baseline level at the end of the experimental period (*P* > 0.05; Fig 4).

**Table 1:** Body composition of the subjects before, after two weeks of detraining and at one, two and three weeks of retraining (mean ± SD).

		Pre	2W DT	1W RT	2W RT	3W RT
Body weight (kg)	HAT	68.1±7.1	68.5±7.1	68.6±7.2	68.6±7.3	68.4±7.3
	DHAT	67.5±7.3	67.8±7.3	67.9±7.3	68.2±7.2	67.9±7.3
Body mass index (kg/m^2^)	HAT	22.7±0.4	22.7±0.5	22.7±0.5	22.5±0.6	22.9±0.4
	DHAT	22.1±0.8	22.4±0.9	22.4±0.6	22.5±0.9	22.3±0.8
Skeletal muscle mass (kg)	HAT	32.6±3.5	32.5±3.3	32.3±3.2	32.6±3.6	32.4±3.3
	DHAT	33.0±3.5	33.2±2.3	33.1±3.2	33.4±3.3	33.3±3.3
Percent body fat (%)	HAT	9.6±0.7	9.7±0.7	9.8±1.3	9.5±0.9	9.9±1.3
	DHAT	9.3±1.2	9.8±1.3	9.8±1.1	9.7±1.2	9.5±1.4

Values are means ± standard deviation

**Table 2:** Differences in the body composition of the subjects between conditions in each test (n = 20).

		F	*P*	$\eta_{P}^{2}$
Body weight	Condition	0.048	0.831	0.005
	Time	12.372	0.001	0.579
	Condition x Time	0.628	0.646	0.065
Body mass index	Condition	2.524	0.147	0.219
	Time	0.716	0.587	0.074
	Condition x Time	1.776	0.155	0.165
Skeletal muscle mass	Condition	0.228	0.644	0.025
	Time	0.178	0.948	0.019
	Condition x Time	0.117	0.976	0.013
Percent body fat	Condition	0.046	0.834	0.005
	Time	2.201	0.088	0.197
	Condition x Time	0.653	0.629	0.068

F; testing criteria level, *P*; level of statistical significance, $\eta_{P}^{2}$; partial eta squared

**Table 3:** Sprint and agility before, after two weeks of detraining and at one, two and three weeks of retraining (mean ± SD).

		Pre	2W DT	1W RT	2W RT	3W RT
5 m	HAT	1.04±0.04	1.05±0.03	1.06±0.04	1.02±0.05	1.02±0.05
	DHAT	1.05±0.04	1.04±0.03	1.05±0.03	1.01±0.04	1.01±0.04
10 m	HAT	1.75±0.06	1.74±0.10	1.73±0.05	1.71±0.04	1.72±0.06
	DHAT	1.78±0.05	1.73±0.05	1.73±0.05	1.71±0.06	1.72±0.07
20 m	HAT	3.00±0.09	3.01±0.13	3.02±0.08	2.99±0.06	2.99±0.09
	DHAT	3.05±0.05	3.07±0.08	3.03±0.06	2.99±0.09	2.99±0.09
30 m	HAT	4.13±0.11	4.22±0.17	4.25±0.12	4.21±0.11	4.23±0.13
	DHAT	4.23±0.07	4.30±0.12	4.29±0.09	4.23±0.12	4.25±0.10
Agility (R)	HAT	8.04±0.19	8.09±0.22	8.13±0.17	7.99±0.21	8.03±0.22
	DHAT	8.06±0.16	8.09±0.22	8.09±0.19	8.04±0.25	8.05±0.25
Agility (L)	HAT	7.99±0.17	8.12±0.20	8.10±0.20	7.98±0.23	8.00±0.18
	DHAT	8.04±0.18	8.14±0.24	8.14±0.18	8.08±0.15	8.08±0.20

Values are means ± standard deviation. R; right, L; left

**Table 4:** Differences in sprint and agility between conditions in each test (n = 20).

		F	*P*	$\eta_{P}^{2}$
5 m	Condition	0.095	0.765	0.010
	Time	7.657	0.001	0.460
	Condition x Time	1.586	0.199	0.150
10 m	Condition	0.305	0.594	0.033
	Time	4.672	0.004	0.342
	Condition x Time	1.010	0.415	0.101
20 m	Condition	0.480	0.506	0.051
	Time	2.500	0.060	0.217
	Condition x Time	1.167	0.342	0.115
30 m	Condition	0.879	0.373	0.089
	Time	5.357	0.002	0.373
	Condition x Time	1.619	0.191	0.152
Agility (R)	Condition	0.013	0.912	0.001
	Time	2.516	0.058	0.218
	Condition x Time	0.357	0.838	0.038
Agility (L)	Condition	0.499	0.498	0.053
	Time	3.542	0.015	0.282
	Condition x Time	0.382	0.820	0.041

R; right, L; left, F; testing criteria level, *P*; level of statistical significance, $\eta_{P}^{2}$; partial eta squared

**Table 5:** Peak torques (Nm) during concentric knee flexion and extension before, after two weeks of detraining and at one, two and three weeks of retraining (mean ± SD).

		Pre	2W DT	1W RT	2W RT	3W RT
DL-PT-E-60	HAT	208.2±12.3	208.2±12.3	209.6±27.3	210.0±25.2	205.2±19.8
	DHAT	211.5±13.9	212.7±15.9	214.2±15.9	216.7±17.5	208.3±12.4
DL-PT-F-60	HAT	135.8±32.3	135.8±30.6	135.8±37.7	139.2±29.5	135.2±28.2
	DHAT	121.2±21.6	137.2±26.9	140.3±29.1	137.2±27.7	136.5±19.1
NL-PT-E-60	HAT	189.9±33.1	194.6±39.0	197.0±35.2	200.9±38.1	191.0±31.0
	DHAT	198.1±28.0	189.1±24.2	183.9±26.8	193.0±24.6	187.0±16.4
NL-PT-F-60	HAT	129.1±30.5	127.9±26.4	125.1±34.8	128.1±26.1	127.1±31.5
	DHAT	132.6±23.1	127.1±20.2	125.7±23.1	131.9±24.6	135.3±26.2
DL-PT-E-180	HAT	138.8±18.9	146.6±21.4	146.6±23.9	145.3±23.3	140.4±18.8
	DHAT	145.2±23.6	146.1±15.1	152.0±20.1	150.3±15.4	152.6±18.8
DL-PT-F-180	HAT	105.4±17.3	105.2±14.8	108.5±13.8	107.3±10.9	107.7±13.4
	DHAT	108.5±9.8	107.2±14.2	110.6±15.7	110.5±17.3	106.1±15.3
NL-PT-E-180	HAT	136.9±18.9	137.0±25.4	139.5±21.1	141.1±18.0	137.7±19.5
	DHAT	138.1±16.5	139.6±16.6	137.9±21.9	142.7±21.4	138.5±20.8
NL-PT-F-180	HAT	97.4±18.7	93.7±18.4	99.7±24.4	96.4±18.9	95.7±21.6
	DHAT	102.6±16.7	100.5±15.3	100.7±19.2	101.7±17.5	102.6±19.9
DL-PT-E-240	HAT	114.9±19.0	115.9±15.9	116.9±17.6	115.2±15.6	113.4±17.1
	DHAT	116.2±16.2	118.6±12.4	117.5±13.6	119.5±12.7	115.8±11.7
DL-PT-F-240	HAT	84.6±15.1	83.8±16.8	84.8±14.1	87.3±12.2	86.2±13.8
	DHAT	89.1±9.3	87.8±13.4	92.6±17.4	92.9±16.0	89.3±12.7
NL-PT-E-240	HAT	113.3±16.7	112.7±15.4	112.6±13.7	114.4±11.2	110.3±14.6
	DHAT	115.2±10.8	110.3±13.1	116.1±14.6	114.2±16.8	115.4±13.2
NL-PT-F-240	HAT	83.3±15.2	83.4±18.6	81.0±19.2	85.1±16.2	86.3±12.8
	DHAT	88.4±14.6	81.7±14.5	86.3±18.4	89.2±18.6	87.2±18.6

Values are means ± standard deviation. DL; dominant leg, NL; non-dominant leg, PT; peak torque, E; extensors, F; flexors, 60, 180, 240; angular velocities (°·s^-1^)

**Table 6:** Differences in sprint and agility peak torques (Nm) during concentric knee flexion and extension between conditions in each test (n = 20).

		F	*P*	$\eta_{P}^{2}$
DL-PT-E-60	Condition	1.674	0.228	0.157
	Time	1.665	0.180	0.156
	Condition x Time	0.100	0.982	0.011
DL-PT-F-60	Condition	0.032	0.862	0.004
	Time	1.249	0.308	0.122
	Condition x Time	1.156	0.346	0.114
NL-PT-E-60	Condition	0.113	0.744	0.012
	Time	0.721	0.583	0.074
	Condition x Time	1.050	0.395	0.104
NL-PT-F-60	Condition	0.059	0.814	0.007
	Time	1.287	0.293	0.125
	Condition x Time	0.469	0.758	0.050
DL-PT-E-180	Condition	0.355	0.566	0.038
	Time	1.283	0.295	0.125
	Condition x Time	0.695	0.600	0.072
DL-PT-F-180	Condition	0.143	0.714	0.016
	Time	0.419	0.794	0.045
	Condition x Time	0.268	0.896	0.029
NL-PT-E-180	Condition	0.007	0.935	0.001
	Time	0.485	0.747	0.051
	Condition x Time	0.177	0.949	0.019
NL-PT-F-180	Condition	0.373	0.556	0.040
	Time	0.481	0.749	0.051
	Condition x Time	0.520	0.721	0.055
DL-PT-E-240	Condition	0.097	0.762	0.011
	Time	0.549	0.701	0.057
	Condition x Time	0.153	0.960	0.017
DL-PT-F-240	Condition	0.872	0.375	0.088
	Time	0.834	0.512	0.085
	Condition x Time	0.297	0.878	0.032
NL-PT-E-240	Condition	0.047	0.833	0.005
	Time	0.651	0.630	0.067
	Condition x Time	0.971	0.435	0.097
NL-PT-F-240	Condition	0.147	0.711	0.016
	Time	1.786	0.153	0.166
	Condition x Time	0.696	0.600	0.072

DL; dominant leg, NL; non-dominant leg, PT; peak torque, E; extensors, F; flexors, 60, 180, 240; angular velocities (°·s^-1^), F; testing criteria level, *P*; level of statistical significance, $\eta_{P}^{2}$; partial eta squared

## Discussion

The major findings in the present study were that two weeks of detraining after competitive season decreased performance in the Yo-Yo IR2 test and repeated sprints. The detraining-induced reductions in the Yo-Yo IR2 test performance improved compared to baseline levels after two weeks of high-intensity aerobic training. Meanwhile, three weeks were required to return to the initial level of repeated sprint performance with retraining using high-intensity training. Ultimately, a reduced amount of high-intensity training after the competitive season facilitated the maintenance of physical fitness.

The HAT group that continued to perform high-intensity aerobic exercise after the competitive season maintained their performance level in the Yo-Yo IR2 test over the five week treatment period. These results are supported by previous studies, which indicate that, after the last match of the season, 10 training sessions, consisting of high-intensity training for two weeks, do not change performance in the Yo-Yo IR2 test in elite soccer players [15]. However, Nakamura et al. [23] observed that running and plyometric training for two days per week for three weeks after the completion of a competitive season did not prevent the decrease in performance in The Yo-Yo IR2 test in elite soccer players. The reason of these differences in results is unclear but it probably related to exercise intensity. Indeed, there was no significant decrease in performance in the Yo-Yo IR2 test during off-season in the present study and Christensen et al. [15]’s study applying high-intensity exercise despite reduced exercise time compared to that in-season. Furthermore, the exercise intensity was higher than that in the previous study conducted by Nakamura et al. [23], which modulated endurance training (70–80% of HRmax).

In the present study, we found that two weeks of detraining after the competitive season markedly decreased performance in the Yo-Yo IR2 test in elite soccer players. Accordingly, Thomassen et al. [5] and Christensen et al. [15] observed that the Yo-Yo IR2 test performance after detraining for two weeks decreased from 845 m to 654 m in elite soccer players. In addition, a study from another laboratory reported that a prolonged detraining period can induce an 8% decline in maximal oxygen consumption [24], which is strongly associated with distance on the Yo-Yo IR2 test [25]. The degree of deterioration of physical fitness over the course of the detraining period after the competitive season is closely related to the fitness level of athletes [23]. Therefore, these results can support the notion that performance in the Yo-Yo IR2 test can be reduced despite only a few days of detraining in elite soccer players with a high level of physical fitness. These decreases in performance in the Yo-Yo IR2 test can be explained at the muscle level. Several weeks of detraining lead to a return in muscle capillarization to baseline before detraining in athletes and a 25%-45% decline in oxidative enzyme activities, which result in reduced mitochondrial ATP production in skeletal muscle [9].

Several previous studies have reported that high intensity training improves the performance in the Yo-Yo IR2 test of elite soccer players [25, 26]. In line with these results, high-intensity aerobic training after two weeks of detraining was found to improve performance in the Yo-Yo IR2 test in the present study. Two weeks of retraining with high-intensity exercise is required to return close to the baseline level of performance. This result is inconsistent with a previous study that suggested that athletes with a high fitness level must perform exercise training for a period that is at least twice as long as the resting time period in order to improve their physical fitness to a level of before detraining [24]. The discrepancy in time periods required to return to the physical fitness level at baseline can be due to variations in the length of the detraining period (four weeks versus eight weeks) and the fitness level of the athletes (compared to end of season versus before the Olympic game). Indeed, the performance in the Yo-Yo IR2 test decreased by 11% at the end of the season compared to the start of the season and a 42% increase was observed during the eight weeks of pre-season training [25]. This phenomenon is likely due to accumulated fatigue experienced during the competitive season. This assumption is supported by the finding from the present study that the performance in the Yo-Yo IR2 test was higher at three weeks of post retraining compared to baseline. Furthermore, Noon et al. [20] and Oliver et al. [27] observed that perceptual well-being (e.g., motivation, sleep quality, recovery, appetite, fatigue, stress, muscle soreness, stiffness) deteriorated with an increase in training exposure and accumulated fatigue as the season progressed in elite athletes.

Repeated sprint performance did not change over five weeks of high-intensity training after competitive season in the present study. In contrast to the present study, previous studies reported that two weeks of high-intensity training immediately after the end of season enhanced repeated sprint performance in elite soccer players [5, 15]. These different results may be associated with the high-intensity training method used during the retraining period. Aguiar et al. [28] observed that intermittent training for 12 weeks consisting of 20 minutes per training session resulted in greater improvements in repeated sprint performance than did continuous training. Indeed, the training sessions in the present study largely comprised of high-intensity endurance exercise, whereas the training sessions used in previous studies consisted of five high-intensity aerobic training, including small-sided (4 vs. 4 and 3 vs. 3) soccer drills (8 × 2 min) and five speed endurance training (10–12 × 25–30 s sprints) over the course of two weeks. In other respects, since well-trained athletes are more sensitive to changes in physical fitness with inadequate training intensity and do not easily experience improvements following further training due to the ceiling effect [25], the capacity of repeated sprint performance of the players in the present study might be optimal by the end of the competitive season. This is supported by the observation that repeated sprint performance in players from the present study was similar to previous study conducted with professional soccer players during the competitive season [18].

It is well known that anaerobic exercise performance decreases in highly trained elite soccer players, despite a short period of detraining after the competitive season [9]. There was also a significant decrease in repeated sprint performance over two weeks of detraining after the end of a match in the present study. The detraining-induced decrease in performance gradually increased during the three weeks retraining period. The aerobic high-intensity training-induced increase in repeated sprint performance in the present study is likely to be the result of training-induced biochemical adaptation in skeletal muscles. Thomassen et al. [5] and Christensesn et al. [15] observed that two weeks of high-intensity exercise immediately after the last match of the season enhanced Na^+^-K^+^ pump α_2_-isoform expression by 15%, increased the FXYD1ser68-to-FXYD1 ratio by 27%, increased the level of pyruvate dehydrogenase by 17%, and improved repeated sprint performance. In comparison, cessation of training for two weeks did not affect the expression of Na^+^-K^+^ pump isoform expression and resulted in a reduction of the AB_FXYD1ser68 signal by 18%; decreased pyruvate dehydrogenase level by 17%; a drop in citrate synthase and 3-hydroxyacyl-CoA activity to 12% and 18% of maximal, respectively; and a reduction in performance. However, repeated sprint performance at 3 weeks post-retraining was still lower than the performance recorded at baseline. As mentioned, aerobic high-intensity training is not optimal for improving repeated sprint performance, which represents the capacity for anaerobic exercise performance. On the contrary, improvements in repeated sprint performance through aerobic high-intensity training might be associated with the training period during the preseason. Recently, Teixeira et al. [29] reported that high-intensity aerobic training involving shuttle-run intervals (4 × 4 min) for five weeks during the preseason enhanced repeated sprint ability with increased aerobic performance in elite athletes. When considered, these findings suggest that more than three weeks of high-intensity aerobic training is required to develop repeated sprint performance during preseason in elite players.

The observed lack of changes in body composition and sprint performances (10 m, 20 m, 30 m) for five weeks during the study period in both groups disagrees with previous studies that engaged in more than two weeks of detraining [13, 30]. For example, Koundourakis et al. [13] examined the effect of detraining on exercise performance and body composition in professional soccer players. They observed that prolonged detraining period (six weeks) significantly increased body weight and body fat percentage and reduced maximal oxygen consumption and performances in squat-jump, countermovement-jump, and sprints (10 m, 20 m). These results suggest that a short period of detraining (approximately two weeks) may not lead to changes in body composition and explosive exercise performance in well-trained soccer players. This is supported by findings that there were changes in neither isokinetic strength at any angular speeds in the present study nor squat, vertical jump, or isometric and isokinetic knee force following two weeks detraining in high fitness athletes [31]. A possible explanation for the absence of changes in explosive exercise performance after a short period of detraining is the lack of changes in muscle fiber characteristics. Mujika and Padilla. [9] reported that two weeks of detraining did not alter muscle fiber distribution in well-trained athletes. However, three weeks of detraining after the first half of a competitive season in elite soccer players resulted in changes in skeletal muscle morphology, including a reduction in mean fast twitch (FT) fiber cross-sectional area and reduction in mitochondrial enzyme activities and exercise performance [32]. Taken together, these data suggest that more than two weeks of detraining in elite soccer players could have resulted in a decrease in explosive exercise performance by reduced ATP production in skeletal muscle.

## Conclusions

In conclusion, the findings demonstrate that two weeks of detraining after the competitive season resulted in a marked decrease in performance in the Yo-Yo IR2 test and repeated sprints. To return to a previous level of physical fitness with retraining through high-intensity aerobic training after a period of detraining required a similar period of retraining for performance in Yo-Yo IR2 and/or more periods for repeated sprint performance. The off-season rest period did not result in changes in explosive exercise performances and body composition. Aerobic high-intensity training with reduced training volume after a competitive season can prevent reductions in performances in the Yo-Yo IR2 test and repeated sprints. On the contrary, the decrease in aerobic and anaerobic performance induced by two weeks of detraining was recovered within a few weeks of adequate training during the preseason. Therefore, these findings suggest that elite soccer players can be allowed to take short periods of rest (~2 weeks) without training during the off-season for the release of mental and physical stress that is accumulated throughout the competitive season.

## Supporting information

S1 File Raw data of Figs 3 and 4 and Tables 1, 2, 3, 4, 5 and 6. (XLSX)

## References

[1] Carling C, Bradley P, McCall A, Dupont G. Match-to-match variability in high-speed running activity in a professional soccer team. J Sports Sci. 2016;34(24):2215–23. Epub 2016/05/05. doi: 10.1080/02640414.2016.1176228 . 27144879 10.1080/02640414.2016.1176228

[2] Di Salvo V, Gregson W, Atkinson G, Tordoff P, Drust B. Analysis of high intensity activity in Premier League soccer. Int J Sports Med. 2009;30(3):205–12. Epub 2009/02/14. doi: 10.1055/s-0028-1105950 . 19214939 10.1055/s-0028-1105950

[3] Mohr M, Krustrup P, Bangsbo J. Match performance of high-standard soccer players with special reference to development of fatigue. J Sports Sci. 2003;21(7):519–28. Epub 2003/07/10. doi: 10.1080/0264041031000071182 . 12848386 10.1080/0264041031000071182

[4] Kotzamanidis C, Chatzopoulos D, Michailidis C, Papaiakovou G, Patikas D. The effect of a combined high-intensity strength and speed training program on the running and jumping ability of soccer players. J Strength Cond Res. 2005;19(2):369–75. Epub 2005/05/21. 15903377 10.1519/R-14944.1

[5] Thomassen M, Christensen PM, Gunnarsson TP, Nybo L, Bangsbo J. Effect of 2-wk intensified training and inactivity on muscle Na+-K+ pump expression, phospholemman (FXYD1) phosphorylation, and performance in soccer players. J Appl Physiol (1985). 2010;108(4):898–905. Epub 2010/02/06. doi: 10.1152/japplphysiol.01015.2009 . 20133439 10.1152/japplphysiol.01015.2009

[6] Mara JK, Thompson KG, Pumpa KL, Ball NB. Periodization and physical performance in elite female soccer players. Int J Sports Physiol Perform. 2015;10(5):664–9. Epub 2015/01/23. doi: 10.1123/ijspp.2014-0345 . 25611789 10.1123/ijspp.2014-0345

[7] Jeong TS, Reilly T, Morton J, Bae SW, Drust B. Quantification of the physiological loading of one week of "pre-season" and one week of "in-season" training in professional soccer players. J Sports Sci. 2011;29(11):1161–6. Epub 2011/07/23. doi: 10.1080/02640414.2011.583671 . 21777053 10.1080/02640414.2011.583671

[8] Buchheit M, Racinais S, Bilsborough JC, Bourdon PC, Voss SC, Hocking J, et al Monitoring fitness, fatigue and running performance during a pre-season training camp in elite football players. J Sci Med Sport. 2013;16(6):550–5. Epub 2013/01/22. doi: 10.1016/j.jsams.2012.12.003 . 23332540 10.1016/j.jsams.2012.12.003

[9] Mujika I, Padilla S. Detraining: loss of training-induced physiological and performance adaptations. Part I: short term insufficient training stimulus. Sports Med. 2000;30(2):79–87. Epub 2000/08/31. . 10966148 10.2165/00007256-200030020-00002

[10] Moore RL, Thacker EM, Kelley GA, Musch TI, Sinoway LI, Foster VL, et al Effect of training/detraining on submaximal exercise responses in humans. J Appl Physiol (1985). 1987;63(5):1719–24. Epub 1987/11/01. doi: 10.1152/jappl.1987.63.5.1719 . 3693207 10.1152/jappl.1987.63.5.1719

[11] Izquierdo M, Ibanez J, Gonzalez-Badillo JJ, Ratamess NA, Kraemer WJ, Hakkinen K, et al Detraining and tapering effects on hormonal responses and strength performance. J Strength Cond Res. 2007;21(3):768–75. Epub 2007/08/10. 17685721 10.1519/R-21136.1

[12] Mujika I, Padilla S. Detraining: loss of training-induced physiological and performance adaptations. Part II: Long term insufficient training stimulus. Sports Med. 2000;30(3):145–54. Epub 2000/09/22. . 10999420 10.2165/00007256-200030030-00001

[13] Koundourakis NE, Androulakis NE, Malliaraki N, Tsatsanis C, Venihaki M, Margioris AN. Discrepancy between exercise performance, body composition, and sex steroid response after a six-week detraining period in professional soccer players. PLoS One. 2014;9(2):e87803 Epub 2014/03/04. doi: 10.1371/journal.pone.0087803 . 24586293 10.1371/journal.pone.0087803 PMC3929557

[14] Buchheit M, Morgan W, Wallace J, Bode M, Poulos N. Physiological, psychometric, and performance effects of the Christmas break in Australian football. Int J Sports Physiol Perform. 2015;10(1):120–3. Epub 2014/05/09. doi: 10.1123/ijspp.2014-0082 . 24806508 10.1123/ijspp.2014-0082

[15] Christensen PM, Krustrup P, Gunnarsson TP, Kiilerich K, Nybo L, Bangsbo J. VO2 kinetics and performance in soccer players after intense training and inactivity. Med Sci Sports Exerc. 2011;43(9):1716–24. Epub 2011/02/12. doi: 10.1249/MSS.0b013e318211c01a . 21311360 10.1249/MSS.0b013e318211c01a

[16] Bartlett JD, Close GL, MacLaren DP, Gregson W, Drust B, Morton JP. High-intensity interval running is perceived to be more enjoyable than moderate-intensity continuous exercise: implications for exercise adherence. J Sports Sci. 2011;29(6):547–53. Epub 2011/03/02. doi: 10.1080/02640414.2010.545427 . 21360405 10.1080/02640414.2010.545427

[17] Impellizzeri FM, Marcora SM, Castagna C, Reilly T, Sassi A, Iaia FM, et al Physiological and performance effects of generic versus specific aerobic training in soccer players. Int J Sports Med. 2006;27(6):483–92. Epub 2006/06/13. doi: 10.1055/s-2005-865839 . 16767613 10.1055/s-2005-865839

[18] Abrantes C, Macas V, Sampaio J. Variation in football players’ sprint test performance across different ages and levels of competition. J Sports Sci Med. 2004;3(YISI 1):44–9. Epub 2004/11/01. . 24778553 PMC3990934

[19] Bangsbo J, Iaia FM, Krustrup P. The Yo-Yo intermittent recovery test: a useful tool for evaluation of physical performance in intermittent sports. Sports Med. 2008;38(1):37–51. Epub 2007/12/18. . 18081366 10.2165/00007256-200838010-00004

[20] Noon MR, James RS, Clarke ND, Akubat I, Thake CD. Perceptions of well-being and physical performance in English elite youth footballers across a season. J Sports Sci. 2015;33(20):2106–15. Epub 2015/09/19. doi: 10.1080/02640414.2015.1081393 . 26383605 10.1080/02640414.2015.1081393

[21] Kilinc BE, Kara A, Camur S, Oc Y, Celik H. Isokinetic dynamometer evaluation of the effects of early thigh diameter difference on thigh muscle strength in patients undergoing anterior cruciate ligament reconstruction with hamstring tendon graft. J Exerc Rehabil. 2015;11(2):95–100. Epub 2015/05/12. doi: 10.12965/jer.150100 . 25960982 10.12965/jer.150100 PMC4415756

[22] Hopkins WG, Marshall SW, Batterham AM, Hanin J. Progressive statistics for studies in sports medicine and exercise science. Med Sci Sports Exerc. 2009;41(1):3–13. Epub 2008/12/19. doi: 10.1249/MSS.0b013e31818cb278 . 19092709 10.1249/MSS.0b013e31818cb278

[23] Nakamura D, Suzuki T, Yasumatsu M, Akimoto T. Moderate running and plyometric training during off-season did not show a significant difference on soccer-related high-intensity performances compared with no-training controls. J Strength Cond Res. 2012;26(12):3392–7. Epub 2011/12/31. doi: 10.1519/JSC.0b013e3182474356 . 22207263 10.1519/JSC.0b013e3182474356

[24] Godfrey RJ, Ingham SA, Pedlar CR, Whyte GP. The detraining and retraining of an elite rower: a case study. J Sci Med Sport. 2005;8(3):314–20. Epub 2005/10/27. . 16248472 10.1016/s1440-2440(05)80042-8

[25] Krustrup P, Mohr M, Nybo L, Jensen JM, Nielsen JJ, Bangsbo J. The Yo-Yo IR2 test: physiological response, reliability, and application to elite soccer. Med Sci Sports Exerc. 2006;38(9):1666–73. Epub 2006/09/09. doi: 10.1249/01.mss.0000227538.20799.08 . 16960529 10.1249/01.mss.0000227538.20799.08

[26] Mohr M, Krustrup P. Comparison between two types of anaerobic speed endurance training in competitive soccer players. J Hum Kinet. 2016;51:183–92. Epub 2017/02/06. doi: 10.1515/hukin-2015-0181 . 28149381 10.1515/hukin-2015-0181 PMC5260561

[27] Oliver JL, Lloyd RS, Whitney A. Monitoring of in-season neuromuscular and perceptual fatigue in youth rugby players. Eur J Sport Sci. 2015;15(6):514–22. Epub 2015/09/15. doi: 10.1080/17461391.2015.1063700 . 26366619 10.1080/17461391.2015.1063700

[28] Aguiar M, Abrantes C, Maçãs V, Leite N, Sampaio J, Ibáñez S. Effects of intermittent or continuous training on speed, jump and repeated-sprint ability in semi-professional soccer players. The Open Sports Sciences Journal. 2008;1:15–9.

[29] Teixeira AS, Arins FB, De Lucas RD, Carminatti LJ, Dittrich N, Nakamura FY, et al Comparative effects of two interval shuttle-run training modes on physiological and performance adaptations in female professional futsal players. J Strength Cond Res. 2017 Epub 2017/09/14. doi: 10.1519/JSC.0000000000002186 . 28902113 10.1519/JSC.0000000000002186

[30] Ostojic SM. Seasonal alterations in body composition and sprint performance of elite soccer players. Journal of Exercise physiology online. 2003;6(3).

[31] Hortobagyi T, Houmard JA, Stevenson JR, Fraser DD, Johns RA, Israel RG. The effects of detraining on power athletes. Med Sci Sports Exerc. 1993;25(8):929–35. Epub 1993/08/01. . 8371654

[32] Bangsbo J, Mizuno M. Morphological and metabolic alterations in soccer players with detraining and retraining and their relation to performance1988.

